# Molecular and morphometric changes in the small intestine during hot and cold exposure in thermally manipulated broiler chickens

**DOI:** 10.14202/vetworld.2021.1511-1528

**Published:** 2021-06-15

**Authors:** Khaleel Emad Khaleel, Mohammad Borhan Al-Zghoul, Khaled Musa Mohammad Saleh

**Affiliations:** 1 Department of Basic Medical Veterinary Sciences, Faculty of Veterinary Medicine, Jordan University of Science and Technology, Jordan; 2 Department of Applied Biological Sciences, Faculty of Science and Art, Jordan University of Science and Technology, Jordan

**Keywords:** broilers, heat shock proteins, immune response, junctional proteins, thermal manipulation, thermal stress

## Abstract

**Background and Aim:**

Thermal stress (hot or cold) is one of many environmental stressors that severely affects the health of broiler chickens. One negative effect of thermal stress is the disruption of the intestinal barrier function in broiler chickens. This study aimed to evaluate the effect of thermal manipulation (TM) on the small intestine in terms of histomorphometry as well as junctional, heat-shock, and immune response gene expression during post-hatch exposure to thermal stress.

**Materials and Methods:**

The experiment was conducted by dividing 928 fertile Ross eggs into three incubation groups: The control (C) group (incubated at 37.8°C and 56% relative humidity [RH] for the whole incubation period), the TM using low temperature TML group (incubated at 36°C and 56% RH for 18 h/day from embryonic days 7 to 16), and the TM using high temperature (TMH) group (incubated at 39°C and 65% RH for 18 h/day from embryonic days 7 to 16). On post-hatch day 21, 90 chicks were randomly selected from each incubation group and were equally subdivided into three subgroups for the post-hatch thermal stress experiment: The TN subgroup (room temperature maintained at 24°C), the heat stress (HS) subgroup (room temperature maintained at 35°C), and the cold stress (CS) subgroup (room temperature maintained at 16°C). After 1 day of thermal stress exposure (age 22 days), five birds from each subgroup were euthanized and ileum samples were collected to evaluate the transcription of the *Claudin* (*CLDN1*)*, CLDN-5, Occludin, Cadherin-1, heat shock factors* (*HSF1*)*, HSF3, 70 kilodalton heat shock protein, 90 kilodalton heat shock protein, Interleukin* *6* (*IL6*)*, IL8, toll-like receptors-2* (*TLR2*), *and TLR4* genes by Real-Time Quantitative Reverse Transcription polymerase chain reaction analysis. Finally, after 4 and 7 days of thermal stress (age 25 and 28 days, respectively), nine chicks were euthanized, and their jejunum and ileum were collected for histomorphometric analysis.

**Results:**

After exposure to 1 day of thermal stress, the C subgroups exposed to thermal stress (HS and CS) possessed significantly increased expression of junctional, heat-shock, and immune response genes compared to the C-TN subgroup, and similar results were observed for the TMH. In contrast, thermally stressed TMH subgroups had significantly lower expression of the studied genes compared to C subgroups exposed to thermal stress. Furthermore, no significant changes were detected between the TML subgroups exposed to thermal stress and TML-TN. Moreover, significant alterations in villus height (VH), villus surface area, crypt depth (CD), and VH to CD ratio were observed between the TML, TMH, and C subgroups exposed to CS.

**Conclusion:**

It might be suggested that TM may have a protective impact on the small intestine histomorphometry and epithelial integrity of broilers during post-hatch exposure to thermal stress.

## Introduction

The exposure of broiler chickens to non-optimal ambient temperatures is considered to be a kind of stress, as the ideal temperature range for broilers, that is, the thermoneutral zone, lies within the range of 18-22°C [1]. The exposure of chickens to an ambient temperature that is significantly higher or lower than that of the thermoneutral zone results in a thermal stress response, a term which refers to the non-organized physiological responses to extreme ambient temperatures that occur to maintain the body temperature (BT) within the ideal range for that organism [1,2]. Thermal stress arises due to the imbalance between the amount of net energy released from the body and the total heat produced by the body’s metabolic processes [2,3]. Subsequently, thermal stress leads to a severe impact on poultry health and production, resulting in commercial losses that are exacerbated during the summer months [2,4-8].

The gastrointestinal tract is the largest part of the body that comes into contact with the external environment, and a sound intestinal barrier is integral in protecting the body from external threats [9]. The protective elements of the intestinal barrier are rooted in tight junction proteins, such as claudins (*CLDN*) and occludins (*OCLN*) as well as adherens junction proteins, such as cadherin (*CDH*) [10]. Any damage to junctional proteins increases the permeability of the intestinal barrier to luminal antigens and may facilitate the translocation of pathogens across it, resulting in endogenous infection and eventual endotoxemia that impairs the absorption of nutrients [11,12]. The previous studies had reported that thermal stress could affect intestinal integrity by modulating the expression of junction and heat shock proteins in the small intestine of broiler chickens and subsequently increasing the expression of pro-inflammatory cytokines and toll-like receptors (TLR) [13-20]. In addition, thermal stress was reported to have severe effect on a broiler’s nutrient absorption capability by modifying the intestine’s histomorphometric parameters, leading to intestinal tissue injury [21-25].

Under thermal stress conditions, the cells respond to the extreme temperatures through a physiological response called heat shock response, which is a specific molecular response to acclimate to the extreme temperature [26]. Heat shock response is mediated through the upregulation and activation of a family of transcription factors called “heat shock factors (HSF),” such as *HSF1* and *HSF3*, which mainly regulates the transcription of HSPs such as 70 kilodalton heat shock protein (*HSP70*) and 90 kilodalton heat shock protein (*HSP90*) [27,28]. The most studied HSP in terms of cellular protection from heat stress (HS) is *HSP70*, as it can be considered to be anHS index in many organisms [29]. HSP possess a critical role in the cell, by their protection of denatured cellular proteins, since HSP correct the folding of damaged proteins, confer protection, prevent formation of protein aggregates, and promote cellular viability [30-32].

During embryonic development, manipulating the incubation temperature was suggested to improve post-hatch thermotolerance acquisition in a process commonly referred to as thermal manipulation (TM) [33-37]. TM enhances thermotolerance by epigenetic thermal adaptation, as the determination of the physiological control center’s set point is highly affected by incubation temperature [38,39]. Previously, TM was reported to improve the small intestinal response to post-hatch thermal exposure by altering the expression of nutrient transporter genes as well as those genes involved in heat-shock, oxidative stress, and inflammatory response [40,41]. In addition, continuous TM from embryonic day 11 until hatching day alleviated the effect of post-hatch *Salmonella* Enteritidis infection on the small intestine by resulting in an increased villus height (VH), improved overall intestinal morphology, and enhanced *HSP70* expression in the ileum [42]. However, the impact of cyclic TM (rather than continuous TM) on intestinal morphometry and integrity during post-hatch exposure to thermal stress is still unknown. Therefore, the aim of the present study is to evaluate the impact of cyclic TM for 18 h/day during embryonic days 7-16 on the morphometry of the jejunum and ileum (VH, villus width [VW], villus surface area [VSA], crypt depth [CD], and VH to CD ratio) and on the expression of junctional proteins (*CLDN1*, *CLDN5*, *OCLN*, and *CDH1*), heat shock proteins (*HSP70*, *HSP90*, *HSF1*, and *HSF3*), and immune response genes (*TLR2*, *TLR4*, Interleukin 6 [*IL6*], and *IL8*) broiler ileums during post-hatch exposure to thermal stress.

## Materials and Methods

### Ethical approval

All procedures conducted in this study were approved by the Jordan University of Science and Technology (JUST) Animal Care and Use Committee.

### Study period and location

Blood samples were collected from March 2019 to April 2020 at the Animal House Facility and Molecular Biology and Virology Laboratory, Faculty of Veterinary Medicine, Jordan University of Science and Technology, Jordan.

### Study population and incubation

**Figure-1:**
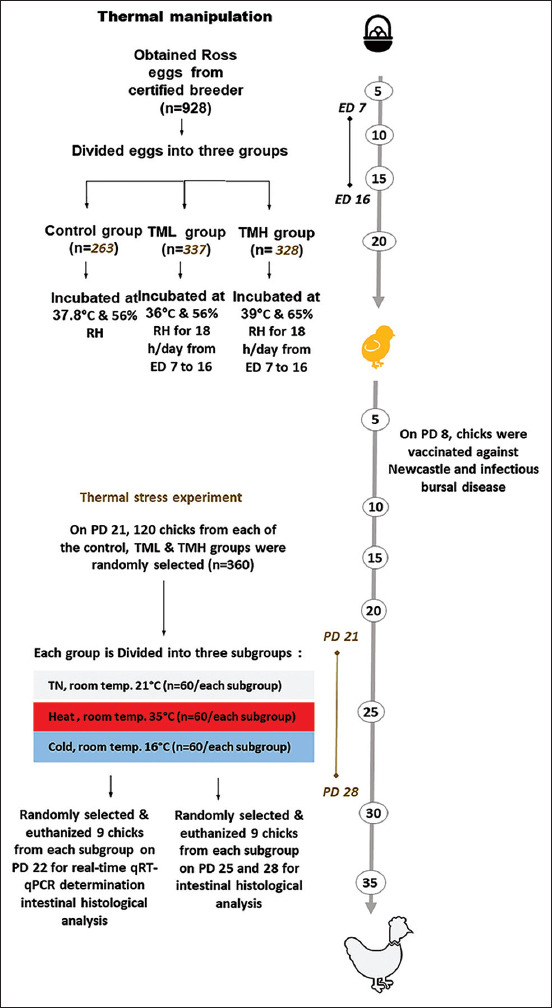
The summary of experimental design executed in the current study. ED=Embryonic day; n=Numbers; PD=Post-hatch day.

Figure-1 shows the summary of the experimental design executed in this study. A total of 1020 fertile eggs from the Ross broiler breed were obtained from certified distributors in Jordan (Amman, Jordan). The eggs were checked for any abnormality or breakage and 92 eggs were excluded. The eggs were divided into three incubation groups: The control group, the TM using low temperature (TML) group, and TM using high temperature (TMH) group. The eggs of the control group were incubated at 37.8°C and 56% relative humidity (RH) throughout the embryogenesis period, while those of the TM groups were incubated as follows: TML group conditions were 36°C and 56% RH for 18 h/day from days 7 to 16 of embryonic development, and TMH group conditions were 39°C and 65% RH for 18 h/day from embryonic days 7 to 16. RH in the TM treatment was elevated to 65% to prevent excessive water loss from the eggs that might result from their previously elevated incubation temperature.

On day 7 of embryonic development, the eggs were examined by candling: The infertile eggs and the eggs with dead embryos were removed. Commercial Type-I HS-SF incubators (Masalles, Barcelona, Spain) were used for egg incubation.

### Hatchery and post-hatch management

On hatching day, the numbers of hatched chicks were recorded every 2 h to measure the early, late, and total hatchability rates. After hatching, the dried chicks were transported to the Animal House at JUST where the field experiments were performed. The chicks were randomly distributed into the cages into groups of 18 chicks per cage pen. Room temperature was maintained at 33±1°C during the 1^st^ week was gradually decreased to 21°C at the beginning of the 4^th^ week and was kept at 21°C until post-hatch day 35. The BT and body weight (BW) were recorded on the following post-hatch days: 1, 4, 5, 7, 14, 16, 18, 20, 21, 26, 30, 32, and 35. Water and appropriate feed were provided to the chicks *ad libitum* throughout the experimental period. The chicks were vaccinated against Newcastle disease and infectious bursal disease on post-hatch day 8.

### Thermal stress experiment

On post-hatch day 21, 120 chicks from each incubation group (control group, TML group, and TMH group) were randomly selected and further subdivided into three subgroups (thermal neutral [TN], cold stress [CS], and HS) to be used in the thermal stress experiment (for a duration of 7 days). For 7 days, TN subgroups were kept at 21°C, CS subgroups were exposed to 16°C, and HS subgroups were exposed to 35°C. On days 1, 4, and 7 of the thermal stress experiments (post-hatch days 22, 25, and 28), nine chicks were randomly selected from each subgroup and their T^b^ and BW were recorded. After 1 day of thermal stress, ileum samples were collected from each subgroup and snap-frozen for subsequent total RNA extraction. Then, the chicks exposed to 4 and 7 days of thermal stress were humanely euthanized to collect jejunum and ileum samples (about 1 cm), which were maintained in a 10% neutral buffered formalin solution for histological morphometric evaluation.

### RNA extraction and cDNA synthesis

Total RNA was extracted from the jejunum and ileum samples using Direct-Zol™ RNA MiniPrep (Zymo Research, Irvine, USA) with TRI Reagent^®^ (Zymo Research, Irvine, USA) according to the protocol of the manufacturer. The RNA was quantified and qualified by Qubit 4 Fluorometer (Thermo Fisher Scientific, MA, USA), and Biotek PowerWave XS2 Spectrophotometer (BioTek Instruments, Inc., USA) and 1% agarose gel. 500 ng of total RNA from each sample was used for cDNA synthesis using Prime Script RT Master Mix kit (Takara Bio Inc., Shiga, Japan).

### Relative mRNA quantitation analysis by real-time qPCR

The QuantiFast SYBR^®^ Green PCR Kit (Qiagen Corp., CA, USA) was used on a Rotor-Gene Q MDx 5 plex platform (QIAGEN Inc., CA, USA). Briefly, the 20 μL reaction mix was prepared by adding 10 μL of master mix, 1 μL forward primer (10 pmol), 1 μL reverse primer (10 pmol), 1 μL cDNA from the sample, and 7 μL of nuclease-free water. The PCR cycles employed the following parameters: 95°C for 5 min; 40 cycles of 95°C for 10 s followed by 30 s annealing (Table-1 for details about annealing temperature); and 72°C for 10 s with final melting at 95°C for 20 s. The detection of fluorescence emission occurred during the extension step. The β-actin gene was used as an internal control to which the fold changes in gene expression were normalized. The single target amplification specificity was approved by the melting curve. The relative quantitation was calculated using 2^−DDCt^ analysis. Table-1 shows primer sequences that were used in the real-time RT-qPCR analysis.

**Table-1:** Primer sequences that are used in the real-time qPCR analysis

Gene	Sequence 5’-3’	Annealing temperature °C
*β-actin*	F: ATGTGGATCAGCAAGCAGGAGTA R: TTTATGCGCATTTATGGGTTTTGT	59
*Claudin-1*	F: CTGATTGCTTCCAACCAG R: CAGGTCAAACAGAGGTACAGG	56
*Claudin-5*	F: CATCACTTCTCCTTCGTCAGC R: GCACAAAGATCTCCCAGGTC	56
*E-cadherin*	F: GACAGGGACATGAGGCAGAA R: GCCGTGACAATGCCATTCTC	57
*HSF1*	F: CAGGGAAGCAGTTGGTTCACTACACG R: CCTTGGGTTTGGGTTGCTCAGTC	63
*HSF3*	F: TCCACCTCTCCTCTCGGAAG R: CAACAGGACTGAGGAGCAGG	57
*HSP70*	F: TCTCATCAAGCGTAACACCAC R: TCTCACCTTCATACACCTGGAC	55
*HSP90*	F: ATGCCGGAAGCTGTGCAAACACAGGACCAA R: GGAATCAGGTTAATTTTCAGGTCTTTTCCA	62
*IL-6*	F: GCTCGCCGGCTTCGA R: GGTAGGTCTGAAAGGCGAACAG	57
*IL-8*	F: CACGTTCAGCGATTGAACTC R: GACTTCCACATTCTTGCAGTG	59
*Occludin*	F: ACGGCAGCACCTACCTCAA R: GGGCGAAGAAGCAGATGAG	59
*TLR-2*	F: CCTGCAACGGTCACTTCAG R: GTCTCAGGGCTTGTTCTTCAG	58
*TLR-4*	F: CTGACCTACCCATCGGACAC R: GCCTGAGAGAGGTCAGGTTG	58

### Histological analysis

After the fixation of the jejunum and ileum in a 10% neutral buffered formalin solution for histological evaluation, the organs were embedded in paraffin, cut to a thickness of 4-5 mm and stained with hematoxylin and eosin stains. Digital images were captured using a B-380 Optika microscope equipped with a C-P6 Pro camera and Optika ProView software (OPTIKA Microscopes, Ponteranica, Italy). Histological examinations were carried out according to the method performed by Shao *et al*. to determine VH, VW, VSA, CD, and VH to CD ratio. VSA was calculated for each villus by multiplying the VH by the VW by p [43].

### Statistical analysis

All statistical analyses were conducted using IBM SPSS statistics software v.25 (IBM software, Chicago, USA). T^b^, BW and *HSP90*, *HSP70*, *HSF1*, *HSF3*, *IL6*, *IL8*, *TLR2*, *TLR4*, *CLDN1*, *CLDN5*, *CDH1*, and *OCLN* mRNA levels were expressed as means±SD. However, VH, VW, VSA, CD, and VH-to-CD ratio were expressed as means±SEM. One-way analysis of variance followed by all-pairs Bonferroni test was used to compare different parameters in all treatment groups, while the Chi-squared test was also used to compare the hatchability rates between the different incubation groups. Parametric differences were considered statistically significant at p<0.05.

## Results

### Impact of TM on broiler hatchability

Table-2 lists the early, late, and total hatchability rates of broiler chickens subjected to embryonic TM. The TML group possessed a significantly lower hatchability rate on embryonic day 20 (p<0.05) and significantly higher hatchability rate on embryonic day 22 compared to the control and TMH groups (p<0.05). In contrast, the TMH group had a significantly higher hatchability rate on embryonic day 20 and a significantly lower one on embryonic day 21 with respect to the control group (p<0.05). However, TM did not have a significant effect on the total hatchability rate.

**Table-2:** Effect of thermal manipulation (TM) during embryogenesis on the hatchability rates of broiler chickens

	Control	TML	TMH
Total eggs	263	337	328
Hatched on ID 20 (hatchability %)	81 (31)^a^	14 (4)^b^	209 (64)^c^
Hatched on ID 21 (hatchability %)	164 (62)^a^	100 (30)^b^	92 (28)^b^
Hatched on ID 22 (hatchability %)	0 (0)^a^	203 (60)^b^	0 (0)^a^
Total hatched	245	317	301
Hatchability %	93.16	94.07	91.77

^a-c^within the same row, values with different superscripts indicate significant differences (P<0.05). ID: incubation day

### Effect of TM on broiler BW

**Figure-2:**
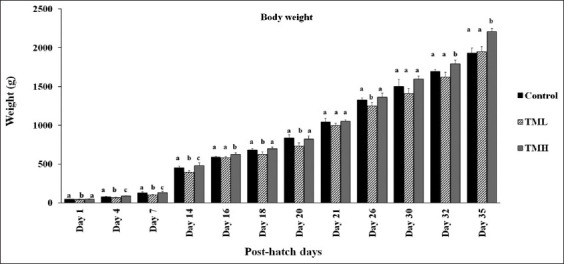
Effects of thermal manipulation (TM) during embryogenesis on post-hatch body weight (BW) of broiler chicks (n=15). ^a-c^ within the same day, means±SD with different superscripts is significantly different (p<0.05).

Figure-2 represents the effect of embryonic TM on the post-hatch BW of broiler chickens. TML significantly decreased BW in all post-hatch days (p>0.05) except for days 16, 21, 30, 32, and 35. Contrastingly, the TMH group exhibited significantly increased BW on post-hatch days 4, 14, 16, 32, and 35 compared to the control group (p>0.05).

### Effect of TM on broiler BT (T^b^)

**Figure-3:**
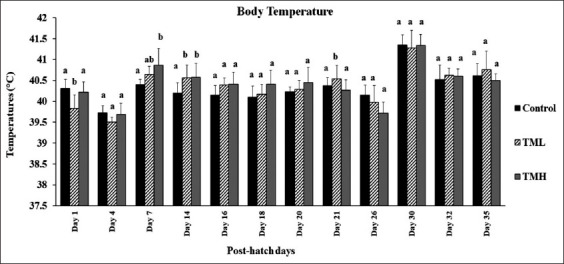
Effects of thermal manipulation (TM) during embryogenesis on post-hatch body temperature (T^b^) of broiler chicks (n=15). ^a,b^ within the same day, means±SD with different superscripts are significantly different (p<0.05).

Figure-3 depicts the effect of embryonic TM on the post-hatch T^b^ of broiler chickens. When compared to the control, the TML group had significantly lower T^b^ on post-hatch day 1 and significantly higher T^b^ on days 14 and 21 (p<0.05). Except for post-hatch days 7 and 14, the T^b^ of TMH group was significantly higher than those of control group (p>0.05); there was no significant difference in T^b^ compared to the other treatment groups.

### Effect of heat and cold exposure on BW of TM broilers

**Figure-4:**
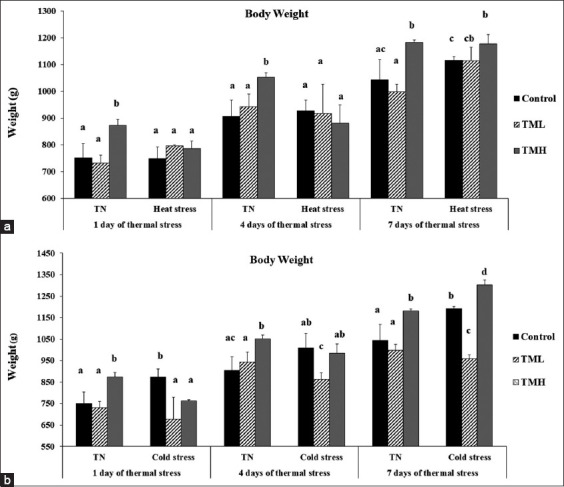
Effects of heat (a) and cold (b) exposures for 7 days (post-hatch days 21-28) on body weight (BW) of broiler chicks subjected to thermal manipulation (TM) during embryogenesis (n=15). ^a-d^ within the same day, means ± SD with different superscripts is significantly different (p<0.05). TN=Thermal neutral, CHS=Chronic heat stress, CCS=Chronic cold stress.

Figure-4a and b depict the effect of heat and cold exposure for 7 days (post-hatch days 21-28) on the BW of TM broilers.

#### TML group

No significant difference was observed in the BW of the TML group compared to those of the control group during heat exposure. However, the BW of the TML group was significantly lower under TN conditions compared to the TML group exposed to 7 days of HS (p<0.05). In addition, under CS, the TML group possessed significantly lower BW compared to the control group exposed to CS and compared to the TML group exposed to TN conditions (p<0.05).

#### TMH group

On the other hand, the BW of the TMH group exposed to HS was significantly lower compared with that of the TMH group exposed to TN conditions after 1 and 4 days of the heat exposure (p<0.05). However, the BW of both groups was not significantly different on the 7^th^ day of HS. Furthermore, under HS, the BW of TMH group was significantly higher than that of control group on the 7^th^ day of heat exposure (p<0.05).

### Effect of heat and cold exposure on BT of TM broilers

**Figure-5:**
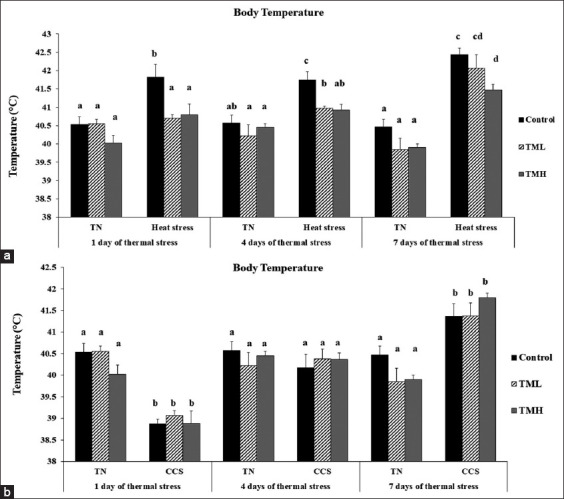
Effects of heat (a) and cold (b) exposures for 7 days (post-hatch days 21-28) on body temperature (T^b^) of broiler chicks subjected to thermal manipulation (TM) during embryogenesis (n=15). ^a-d^ within the same day, means±SD with different superscripts is significantly different (p<0.05). TN=Thermal neutral, CHS=Chronic heat stress, CCS=Chronic cold stress.

Figure-5a and b depict the effect of heat and cold exposure for 7 days (post-hatch days 21-28) on the BT of TM broilers.

#### TML group

Under HS, the TML group had a significantly lower BT than the control group on days 1 and 4 of heat exposure (p<0.05). In addition, the TML group exposed to HS had significantly higher BT than the TML group kept under TN conditions (p<0.05). Under cold exposure, the TML group did not show a significant difference in BT compared to the control group. However, the TML group exposed to CS possessed significantly lower BT than the TML group kept under TN conditions on day 1 of cold exposure, but it was significantly higher on day 7 of cold exposure (p<0.05).

#### TMH group

The BT of the TMH group was significantly higher on day 7 of heat exposure compared to the TMH group exposed to TN condition (p<0.05). However, the BT of the TMH group was significantly lower than that of the control group under HS conditions (p<0.05). No significant difference in BT was detected between the TMH and control groups exposed to CS. In contrast, compared with the TMH group kept under TN condition, the TMH group exposed to CS possessed significantly lower BT after 1 day and significantly higher BT after 7 days of cold exposure (p<0.05).

### Effect of heat and cold exposure on the mRNA Levels of junctional proteins in the ileum of TM broilers

**Figure-6:**
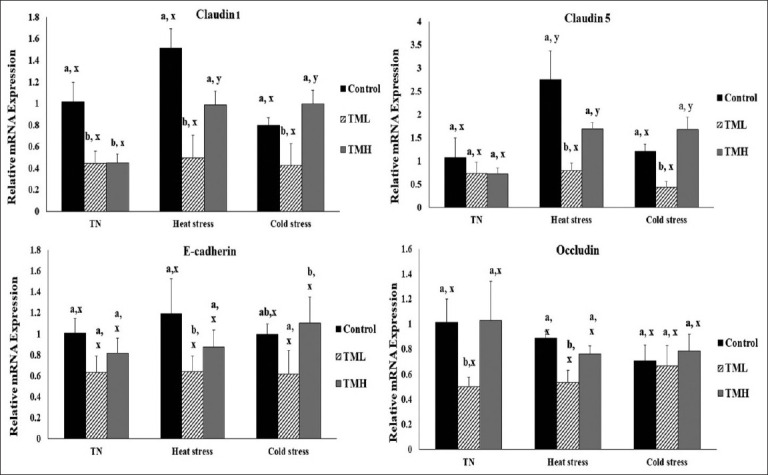
Effects of heat and cold exposures for 1 day on the mRNA levels of junctional proteins (*CLDN*, *CLDN*, *E*-*CDH*, and *OCLN*) in the ileum of broiler chicks subjected to thermal manipulation (TM) during embryogenesis (n=5). The values in the chart indicate folds of mRNA level in the control group-TN. ^a,b^ within the same condition (either TN, heat or cold stress) and between different incubation groups (control, TML, and TMH), means ± SD with different superscripts are significantly different (p<0.05). ^x,y^ within the same incubation group but between TN, heat, and cold exposures, means ± SD with different superscripts are significantly different (p<0.05).

Figure-6 depicts the effect of 1-day heat and cold exposure on the mRNA levels of the *CLDN1*, *CLDN5*, *CDH1*, and *OCLN* genes in the ileum of TM broiler chicks.

#### CLDN1

Under TN, heat, and cold exposure, *CLDN1* expression was significantly decreased in the TML group compared to the control (p<0.05). In addition, the TMH group led to a significantly lower *CLDN1* expression compared to the controls only during TN condition (p<0.05). However, the TMH groups exposed to heat and cold exposures exhibited significantly higher *CLDN1* expression compared to the TMH group kept under TN condition (p<0.05). In contrast, *CLDN1* expression was not significantly different among TML groups under TN, heat, and cold exposures.

#### CLDN5

The TML group had significantly lower *CLDN5* expression compared to the control group under both heat and cold exposures (p<0.05), while no significant difference was observed in *CLDN5* expression of the TMH group compared to the control. On the other hand, the control group exposed to HS had significantly higher *CLDN5* expression than the control group kept under TN condition (p<0.05). Moreover, TMH groups under heat and cold exposures showed significantly higher *CLDN5* expression compared to the TMH group kept under TN condition (p<0.05). However, no significant change was detected in *CLDN5* expression between the TML groups under TN, heat, and cold exposures.

#### CDH1

Under HS, the TML group had significantly lower *CDH1* expression compared to the control group (p<0.05), but under TN and cold exposures, no significant difference between the TML and control groups was detected (p>0.05). Furthermore, no significant change in the *CDH1* expression was detected between the TMH and control groups under TN, heat, and cold exposures.

#### OCLN

*OCLN* expression under TN and HS conditions was significantly lower in the TML group compared with the control group (p<0.05), while no significant difference was observed between the TML and control groups under cold exposure. Moreover, no significant difference was observed between the TMH and control groups under TN, heat, and cold exposures.

### Effect of heat and cold exposure on the mRNA levels of heat shock proteins in the ileum of TM broilers

**Figure-7:**
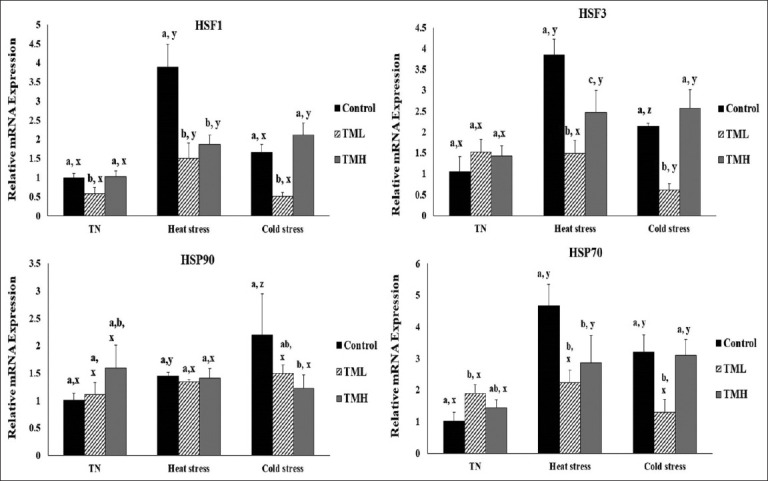
Effects of heat and cold exposures for 1 day on the mRNA levels of heat shock proteins (*HSF1*, *HSF3*, HSP90, and *HSP70*) in the ileum of broiler chicks subjected to thermal manipulation (TM) during embryogenesis (n=5). The values in the chart indicate folds of mRNA level in the control group-TN. ^a-c^ within the same condition (either TN, heat, or cold stress) and between different incubation groups (control, TML, and TMH), means ± SD with different superscripts is significantly different (p<0.05). ^x,z^ within the same incubation group but between TN, heat, and cold exposures, means ± SD with different superscripts are significantly different (p<0.05).

Figure-7 depicts the effect of 1-day heat and cold exposure on the mRNA levels of heat shock proteins (*HSF1*, *HSF3*, *HSP90*, and *HSP70*) in the ileum of TM broiler chicks.

#### HSF1

Under TN, heat, and CS conditions, the TML group exhibited significantly lower *HSF1* expression compared to the control group (p<0.05). The TMH group showed significantly lower *HSF1* expression compared to controls under heat exposure (p<0.05), while no difference between them was detected under TN and cold exposures (p>0.05). In addition, the control group kept under TN condition had significantly lower *HSF1* expression than the control group exposed to HS (p<0.05). Similarly, the TML group kept under TN conditions possessed significantly lower *HSF1* expression than the TML group exposed to HS (p<0.05). Furthermore, the TMH group kept under TN conditions had significantly lower *HSF1* expression than the TMH groups under both heat and cold exposures (P<0.05).

#### HSF3

Under both heat and cold exposures, the TML group had significantly lower *HSF3* expression with respect to controls (p<0.05). In contrast, the TMH group possessed significantly lower *HSF3* expression compared to controls only under heat exposure (p<0.05). On the other hand, the control group kept under TN conditions had significantly lower *HSF3* expression compared to control groups exposed to heat and CS (p<0.05), and similar results were observed for the TMH group. However, *HSF3* expression in the TML group exposed to TN conditions was not significantly different from the TML groups exposed to both heat and CS).

#### HSP70

Under TN conditions, the TML group had significantly increased *HSP70* expression compared to the control group (p<0.05). However, under both heat and cold exposures, the TML group had significantly lower *HSP70* expression with respect to controls (p<0.05). Under heat exposure, the TMH group exhibited significantly lower *HSP70* expression compared to controls (p<0.05). However, no significant difference was observed between the TMH and control groups under both TN and cold exposures. On the other hand, control groups exposed to both heat and cold conditions had significantly increased *HSP70* expression compared to the control group kept under TN condition (p<0.05). Similar results were observed for the TMH group, but no significant change in *HSP70* expression was observed between the TML groups under TN, heat, and CS conditions.

#### HSP90

*HSP90* expression was significantly increased in the control group exposed to heat and cold conditions compared to that in the control group kept under TN conditions (p<0.05). However, *HSP90* expression in the TML and TMH groups was not significantly changed between the TN, heat, and cold exposure conditions. Furthermore, *HSP90* expression was significantly lower in the TMH group than in control group under cold exposure (p<0.05).

### Effect of heat and cold exposure on the mRNA levels of immune-related genes in the ileum of TM broilers

**Figure-8:**
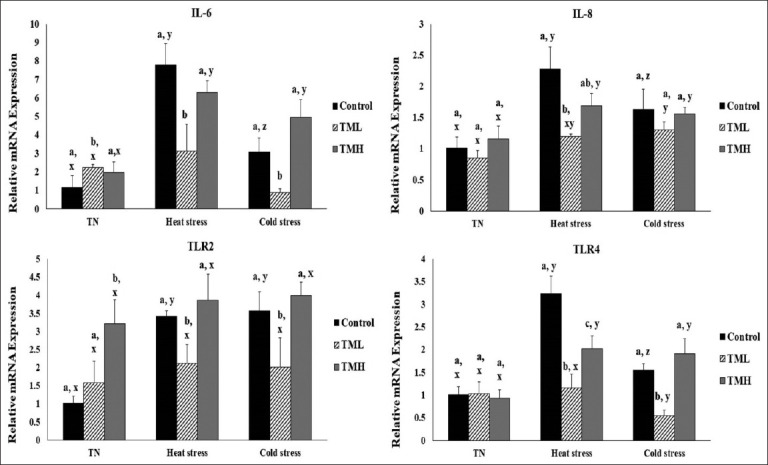
Effects of heat and cold exposures for 1 day on the mRNA levels of toll-like receptors (*TLR2* and *TLR-4*) and pro-inflammatory cytokines (*IL-6* and *IL-8*) in the ileum of broiler chicks subjected to thermal manipulation (TM) during embryogenesis (n=5). The values in the chart indicate folds of mRNA level in the control group-TN. ^a-c^ within the same condition (either TN, heat, or cold stress) and between different incubation groups (control, TML, and TMH), means ± SD with different superscripts is significantly different (p<0.05). ^x,z^ within the same incubation group but between TN, heat, and cold exposures, means ± SD with different superscripts are significantly different (p<0.05).

Figure-8 represents the effect of 1-day heat and cold exposure on the mRNA levels of toll-TLR (*TLR2* and *TLR4*) and pro-inflammatory cytokines (*IL6* and *IL8*) in the ileum of TM broiler chicks.

#### TLR2

*TLR2* expression in the TMH group was significantly higher than that in control group under TN conditions (p<0.05). Under heat and cold exposures, *TLR2* expression in the TML group was significantly lower than that in control group (p<0.05). In addition, *TLR2* expression in the control group was significantly increased under heat and cold exposures compared to that under TN condition (p<0.05).

#### TLR4

Under heat and cold exposures, *TLR4* expression was significantly lower in the TML group than in the control group (p<0.05). Furthermore, *TLR4* expression was significantly lower in the TMH group compared to that in the control group under HS conditions (p<0.05). Moreover, *TLR4* expression in both the TMH and control groups exposed to TN conditions was significantly lower than that in the TMH and control groups under heat and under CS conditions (p<0.05). However, *TLR4* expression in the TML group kept under TN condition was significantly higher than that in TML group exposed to CS (p<0.05).

#### IL6

*IL6* expression in the TML group was significantly lower than that in the control group during both heat and cold exposures (p<0.05). On the other hand, *IL6* expression was significantly lower in the control and TMH groups kept under TN conditions than that in counterpart groups exposed to both heat and CS conditions (p<0.05).

#### IL8

*IL8* expression was significantly lower in the TML group compared to that in the control group during heat exposure (p<0.05). In addition, *IL8* expression was significantly lower in the control and TMH groups kept under TN conditions compared to that in the counterpart groups exposed to heat and cold exposures (p<0.05). Finally, *IL8* expression was significantly lower in the TML group kept under TN condition than that in the counterparts exposed to CS (p<0.05).

### Effect of heat and cold exposures on the morphology of the small intestines of TM broilers

**Figure-9:**
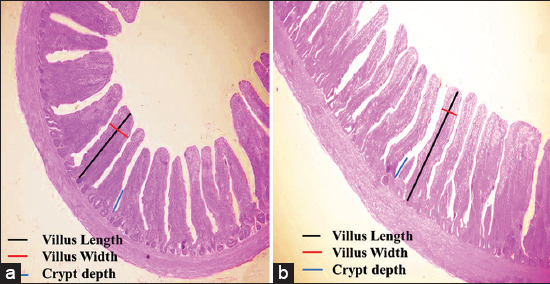
(a and b) Depicts the microscopic measurements of VH, VW, and CD for both jejunum and ileum. VH=Villus height, VW=Villus width, VSA=Villus surface area, CD=Crypt depth, VH: CD=Villus height to crypt depth ratio.

Figure-9 depicts microscopic measurements of VH, VW, and CD for both the jejunum and ileum.

#### Impact of 4-day thermal exposure on ileum morphology

Table-3 represents the effect of 4-day heat and cold exposures (post-hatch days 21-25) on the morphology of the ileum in TM broiler chicks. No significant changes were observed in the VH, VW, VSA, CD, and VH: CD ratio between the different groups in the ileum after 4 days of heat and cold exposure (p>0.05).

**Table-3:** Effects of heat and cold exposures for 4 days (post-hatch days 21-25) on the morphology of ileum in broiler chickens subjected to thermal manipulation (TM) during embryogenesis (n=3).

Day 4 of stress		Ileum				

		VH (µm)	VW (µm)	VSA (µm^2^)	CD (µm)	VH:CD(µm)
TN	Control	600.61 ± 27.43	154.67 ± 7.31	292758 ± 25479	142.99 ± 3.79	4.2 ± 0.16
	TML	649.13 ± 16.34	137.81 ± 16.79	282610 ± 41917	156.58 ± 7.82	4.17 ± 0.26
	TMH	662.73 ± 104.83	135.08 ± 11.57	274235 ± 21247	151.18 ± 17.27	4.34 ± 0.19
CHS	Control	594.75 ± 42.37	137.4 ± 13.16	260095 ± 44391	180.99 ± 19.41	3.42 ± 0.63
	TML	632.01 ± 60.31	157.99 ± 15.27	319131 ± 57304	134.39 ± 11.57	4.82 ± 0.79
	TMH	646.22 ± 40.6	142.74 ± 12.6	291838 ± 39722	144.07 ± 18.02	4.61 ± 0.59
CCS	Control	720.44 ± 63.96	125.42 ± 7.15	286560 ± 409180	167.66 ± 26.82	4.45 ± 0.54
	TML	656.95 ± 86.62	173.02 ± 37.31	361795 ± 108242	170.26 ± 25.56	3.92 ± 0.46
	TMH	892.48 ± 205.18	120.04 ± 3.02	333993 ± 71003	163.72 ± 3.38	5.44 ± 1.21

TN: thermal neutral. CHS: chronic heat stress. CCS: chronic cold stress. VH: villus height. VW: villus width. VSA: villus surface area. CD: crypt depth. VH:CD: villus height to crypt depth ratio

#### Impact of 4-day thermal exposure on jejunum morphology

Table-4 displays the Effects of 4-day heat and cold exposures (post-hatch days 21-25) on the morphology of the jejunum in TM broiler chicks. No significant changes were detected in the VH, VW, VSA, CD, and VH: CD ratio between the control, TML and TMH groups under TN and HS conditions. During CS, the VH, VSA, and VH: CD ratio were significantly higher in the TML and TMH groups exposed to CS compared to that in the TML and TMH groups kept under TN conditions (p<0.05). In addition, significant increases in the VH and CD were observed in the control group exposed to CS with respect to that exposed to TN conditions (p<0.05). Furthermore, under CS, the TMH group had significantly lower CD than the control group, and the TML and TMH groups had significantly higher VH: CD ratio than the control group (p<0.05).

**Table-4:** Effect of heat and cold exposures for 4 days (post-hatch days 21-25) on the morphology of jejunum in broiler chickens subjected to thermal manipulation (TM) during embryogenesis (n=3).

Day 4 of stress		Jejunum				

		VH (µm)	VW (µm)	VSA (µm^2^)	CD (µm)	VH:CD(µm)
TN	Control	835.41 ± 24.03^a^	153.66 ± 7.13	403212 ± 22679.3^a^	148.72 ± 8.37^ac^	5.65 ± 0.3^ac^
	TML	800.59 ± 43.99^a^	140.32 ± 18.18	352530 ± 46415^a^	173.25 ± 8.02^abc^	4.63 ± 0.21^a^
	TMH	971.91 ± 40.86^ac^	135.24 ± 2.87	413384 ± 25910^a^	192.64 ± 29.63^abc^	5.28 ± 0.78^a^
CHS	Control	888.19 ± 59.23^ac^	147.3 ± 1.4	411339 ± 31237^a^	202.1 ± 7.75^abc^	4.39 ± 0.13^a^
	TML	832.8 ± 46.03^ac^	153.91 ± 19.74	397870 ± 34897^a^	228 ± 29.01^ab^	3.73 ± 0.32^a^
	TMH	1111 ± 76.38^bc^	145.78 ± 18.89	500007 ± 38234^ac^	205.46 ± 11.27^abc^	5.4 ± 0.08^a^
CCS	Control	1115.71 ± 54.7^bc^	170.36 ± 22.87	604126 ± 111257^ab^	240.31 ± 10.33^b^	4.66 ± 0.33^a^
	TML	1305.68 ± 86.43^b^	198.41 ± 6.19	812706 ± 55858^b^	166.17 ± 15.25^abc^	8.03 ± 1.04^bc^
	TMH	1270.03 ± 6.04^b^	175.36 ± 10.74	699161 ± 41537^bc^	137.41 ± 1.69^c^	9.25 ± 0.15^b^

TN: thermal neutral. CHS: chronic heat stress. CCS: chronic cold stress. VH: villus height. VW: villus width. VSA: villus surface area. CD: crypt depth. VH:CD: villus height to crypt depth ratio. ^a-c^within the same column, means ± SEM with different superscripts are significantly different (p<0.05)

#### Impact of 7-day thermal exposure on ileum morphology

Table-5 represents the Effects of 7-day heat and cold exposures (post-hatch days 21-28) on the morphology of the ileum in TM broiler chicks. No significant changes were observed in the VH, VW, and VSA between the control, TML and TMH groups under different conditions. However, the CD was significantly higher and the VH: CD ratio was significantly lower in the control group exposed to TN conditions than that in the control exposed to heat and cold exposures (p<0.05).

**Table-5:** Effects of heat and cold exposures for 7 days (post-hatch days 21-28) on the morphology of ileum in broiler chickens subjected to thermal manipulation (TM) during embryogenesis (n=3).

Day 7 of stress		Ileum				

		VH (µm)	VW (µm)	VSA (µm^2^)	CD (µm)	VH:CD(µm)
TN	Control	589.46 ± 34.83^ab^	154.66 ± 19.49	286448 ± 39650	165.85 ± 8.22^a^	3.59 ± 0.38^a^
	TML	623.96 ± 24.58^ab^	160.99 ± 8.96	316767 ± 29997	160.51 ± 2.71^a^	3.89 ± 0.13^ac^
	TMH	702.51 ± 28.51^a^	153.15 ± 10.35	338045 ± 27402	146.57 ± 4.34^ab^	4.8 ± 0.27^abc^
CHS	Control	612.83 ± 12.7^ab^	146.54 ± 18.22	283321 ± 40641	102.9 ± 1.15^b^	5.96 ± 0.13^b^
	TML	579.02 ± 17.23^b^	128.86 ± 3.59	234214 ± 8768	130.73 ± 19.85^ab^	4.61 ± 0.61^ab^
	TMH	700.37 ± 12.19^a^	146.03 ± 8.62	321582 ± 23228	133.75 ± 11.95^ab^	5.3 ± 0.37^bc^
CCS	Control	605.37 ± 26.47^ab^	162.35 ± 12.57	309887 ± 34408	113.75 ± 6.92^b^	5.33 ± 0.11^bc^
	TML	634.12 ± 19.87^ab^	154.34 ± 15.22	306636 ± 28696	151.83 ± 3.84^ab^	4.18 ± 0.18^ac^
	TMH	704.88 ± 16.89^a^	162.3 ± 8.13	360044 ± 26628	142.67 ± 11.7^ab^	4.99 ± 0.34^ab^

TN: thermal neutral. CHS: chronic heat stress. CCS: chronic cold stress. VH: villus height. VW: villus width. VSA: villus surface area. CD: crypt depth. VH:CD: villus height to crypt depth ratio. ^a-c^within the same column, means ± SEM with different superscripts are significantly different (p<0.05).

#### Impact of 7-day thermal exposure on jejunum morphology

Table-6 represents the Effects of 7-day heat and cold exposures (post-hatch days 21-28) on the morphology of the jejunum in TM broiler chicks. The VH, VW, VSA, and CD did not significantly differ between the control, TML and TMH groups under the different conditions. However, the VH: CD ratio was significantly lower in the TMH group than in the control group under HS conditions (p<0.05).

**Table-6:** Effects of heat and cold exposures for 7 days (post-hatch days 21-28) on the morphology of jejunum in broiler chickens subjected to thermal manipulation (TM) during embryogenesis (n=3).

Day 7 of stress		Jejunum				

		VH (µm)	VW (µm)	VSA (µm^2^)	CD (µm)	VH:CD(µm)
TN	Control	826.93 ± 46.84	154.22 ± 17.19	395423 ± 20616	144.5 ± 17.69	5.95 ± 0.94^ab^
	TML	809.49 ± 72.79	153.6 ± 2.39	390563 ± 36146	167.74 ± 15.92	4.84 ± 0.18^a^
	TMH	972.88 ± 55.1	152.7 ± 15.39	470638 ± 66996	186.53 ± 19.45	5.3 ± 0.46^a^
CHS	Control	1077.55 ± 179.38	127.55 ± 11.57	431846 ± 85276	124.89 ± 19.57	8.6 ± 0.19^b^
	TML	818.61 ± 21.61	145.4 ± 10.59	373906 ± 29725	152.05 ± 15.43	5.51 ± 0.63^ab^
	TMH	912.91 ± 186.41	149.94 ± 8.37	431528 ± 95771	174.2 ± 34.75	5.29 ± 0.82^a^
CCS	Control	999.37 ± 131.72	147.4 ± 14.26	451968 ± 27685	177.68 ± 28.22	5.75 ± 0.6^ab^
	TML	1043.09 ± 105.68	148.26 ± 24.49	470046 ± 35167	195.93 ± 10.14	5.41 ± 0.85^ab^
	TMH	1000.27 ± 20.17	146.97 ± 12.12	460470 ± 30553	146.33 ± 1.78	6.84 ± 0.1^ab^

TN: thermal neutral. CHS: chronic heat stress. CCS: chronic cold stress. VH: villus height. VW: villus width. VSA: villus surface area. CD: crypt depth. VH:CD: villus height to crypt depth ratio. ^a,b^within the same column, means ± SEM with different superscripts are significantly different (p<0.05)

## Discussion

TM by elevating or decreasing the incubation temperature of broiler eggs is postulated to be an important strategy to improve post-hatch thermotolerance acquisition. However, such embryonic TM should be applied during the critical period of hypothalamic-pituitary-thyroid and hypothalamic-pituitary-adrenal axes development [43,44]. TM is used to change the “setpoint” of the systems regulating thermoregulation during the development and maturation of the thermoregulatory center in the brain (e.g., days 6-16 of incubation) [45]. The aim of the current study was to examine the influence of embryonic TM on ileum mRNA levels of junctional proteins (*CLDN1*, *CLDN5*, *OCLN*, and *CDH1*), heat shock proteins (*HSP70* and *HSP90)*, *HSF* (*HSF1* and *HSF3*), and immune response genes (*IL6, IL8, TLR2*, and *TLR4*) as well as on the histological morphometrics of the jejunum and ileum during post-hatch exposure to heat and CSs.

### Impact of TML and TMH temperatures on the physiological parameters of broilers subjected to thermal stress

TMH accelerated the hatchability rate compared with the control group, while the TML group had an insignificant delaying effect on the hatchability. The previous studies reported contradictory findings on the overall hatchability rate, which may be attributed to the use of broilers of different strains types, ages, or incubation temperatures and relative humidities [46-49]. Formerly, a low incubation temperature was found to extend the incubation period of broiler eggs and subsequently delayed hatching [50,51]. This extended incubation period occurred due to the slowing down of the metabolism (hypometabolism), mainly of lipids and carbohydrates [51]. On a similar note, it was shown previously that a high incubation temperature shortens the incubation period due to the increased metabolism [52-55].

The present study showed that TML led to significantly decreased BWs during the early post-hatch life, but no significant difference in the BWs was observed during the 5^th^ week of the post-hatch life. Except for day 7 of CHS, there was significant reduction in the BW of the stressed-TMH groups compared to TN-TMH groups on day 1 and 4 of CHS (p<0.05). Similarly, during CCS, except for day 1 and day 4, there was significant increase in the BW of the stressed-TMH groups compared to TN-TMH groups after 7 days of CCS (p<0.05).

Previously, conflicting results were found for BWs in the early post-hatch life, since different studies reported that TM increased, reduced, or had no impact on hatchling weights [56-59]. The decreased BW during early post-hatch life in TML chicks is mostly associated with the decreased metabolic rate, since a lower metabolic rate could lead to delayed and retarded growth as energy is diverted toward maintenance of existing tissues rather than growth [50]. However, it had been reported that a low incubation temperature led to significantly increased BW during post-hatch life [60]. In the current study, TMH led to significantly higher BW especially in the 5^th^ week post-hatch. Consistently, it had been reported that eggs incubation at 38.5°C on embryonic days 16-18 and at 39°C 18 h/d on embryonic days 12-18 led to increased broiler BW, however, eggs incubation at 39.5°C for 3 h/d during ED 8-10 did not significantly affect poultry BW during post-hatch life [49,61]. Previously, it had been shown that eggs incubation at 39.5°C for 12 h/d on embryonic days 7-16 increased myofiber diameters and improved muscle growth in comparison with controls until post-hatch day 35 [62]. In addition, the cyclic increased incubation of broiler temperature of broiler eggs was reported to increase pectoral muscle mass and increased myoblast proliferation during their post-hatch lives [49,63-65].

No major T^b^ changes were observed at all stages of development, except for the post-hatch days 1, 7, 14, and 21. Similarly, it has been reported that T^b^ of TM chicks (38.8°C for 6 or 18 h) did not differ significantly from that of the controls [37,49]. In the contrary, Loyau *et al*. [66] reported previously that the T^b^ of TM chicks were significantly lower (39.5°C for 12 h during ED 7-16) than those of the controls at hatching and until day 28 of age. Moreover, the previous studies have also reported that TM (39.5°C for 12 h/d during ED 7-16) had a long-lasting effect on thermotolerance acquisition of broilers that persisted until an early marketing age (day 35 of age) [62,67-69]. Those results showed that selected TM conditions have not improved the long-term thermotolerance acquisition in chicks following TM.

Interestingly, the present study shows that the T^b^ of the TM chicks was significantly lower than that of the controls after 1, 4, and 7 days of CHS compared to those of the controls. Our results indicate that TM improves thermotolerance acquisition in TM chicks. Previously, it has been reported that the T^b^ of TM chicks was significantly lower than that of controls [56,66,70]. On the other hand, Collin *et al*. reported significant hyperthermia in TM and control chicks following HS on post-hatch day 42, with higher mortality rates in TM chicks [50]. These results indicate that the TM conditions tested by Collin *et al*. [49] failed to improve long-term thermotolerance acquisition in TM chicks. Furthermore, the previous studies have also reported that TM (39.5°C for 12 h/d during ED 7-16) had a long-lasting effect on thermotolerance acquisition by broilers that persisted until an early marketing age (day 35 of age) [62,67,68]. This suggested that TM during broiler chicken embryogenesis may improve thermotolerance acquisition in chickens raised in regions with high ambient temperatures, with a potentially significant improvement in economic return.

### Impact of TML and TMH temperatures on the expression of junctional and heat shock genes in broilers subjected to thermal stress

Thermal stress (both heat and cold) is one of the serious physical factors that impair the intestinal barrier. The significant damage to the intestinal epithelia is a major cause of the mortality associated with thermal stress [71-75]. Under thermal stress, the body shifts the thermoregulatory mechanisms away from the viscera and toward the peripheral circulation to assist in heat dissipation, which results in visceral ischemia and subsequent hypoxia. Hypoxic conditions in the gastrointestinal tract, which is part of the viscera, lead to epithelial deterioration and dysfunction of junctional proteins in the intestinal barrier [76]. The intestinal barrier is composed of both adherents and tight junctions, and it possesses a critical role in nutrient, water, and electrolyte absorption as well as in the protection of the gastrointestinal tract from pathogenic invasions [77-79].

The tight junction proteins, *CLDN* and *OCLN*, comprise the apical-most junction of a series of cellular contacts that build lateral connections between neighboring cells [80,81]. Any damage in the intestinal junctional proteins increases the permeability to the luminal antigens and pathogens translocation, resulting in endogenous infection and eventually endotoxemia, and impairs the absorption of nutrients [11,12].

The present study illustrated that HS increased *CLDN1* and *CLDN5* expression in the control and TMH groups compared to their TN counterparts. CS also significantly increased *CLDN1* and *CLDN5* expression in the TMH subgroup, but neither heat nor CS affected the expression of these genes in the TML subgroup. In contrast, heat and CS did not significantly change the expression of the *OCLN* and *CDH1* genes in any of the subgroups. Moreover, the expression of all evaluated junctional proteins was significantly lower in the TML subgroup compared with the control subgroups under thermal stress exposure.

Corresponding with our findings, it was found that thermal exposure increases the expression of *CLDN1* [14-16], *OCLN* [16], *CDH1*, and *CLDN5* [14]. These findings suggest that upregulation in gene expression is a compensatory response to the damaged junctional proteins or to the rupture of the intestinal epithelia. However, such a compensatory response was not observed in the TML subgroup as this subgroup possessed comparable mRNA levels of junctional proteins under all tested conditions, suggesting that this subgroup was less affected by thermal stress. Previously, it was found that continuous TM from embryonic day 11 until hatching day improved the overall intestinal morphology after post-hatch exposure to artificial infection with *S. Enteritidis* [42].

In the present study, thermal stress significantly upregulated *HSF1*, *HSF3*, *HSP70*, and *HSP90* expression in the control group. Similar findings were observed in the TMH subgroup (except for *HSP90*), but the expression of these upregulated genes was significantly lower than that in the control subgroup. In contrast, for the TML subgroup, HS only increased *HSF1* expression and CS did not change the expression of any of the investigated heat shock genes.

In accordance with our findings, TM has been found to have a long-lasting effect on *HSF1*, *HSF3*, and *HSP70* expression, which was linked to improved acquisition of heat tolerance in broilers [35,37,70,82]. Such alterations in expression dynamics may be the result of epigenetic changes triggered by TM, as TM was previously found to affect DNA methylation of the *HSP70* promoter region which subsequently altered its expression during post-hatch life [83]. Similarly, it was shown that chicks subjected to TM resulted had decreased *HSF1*, *HSF3*, and *HSP70* expression in the jejunal mucosa after 7 days of HS exposure in comparison with chicks incubated under standard conditions [40]. Furthermore, CS was reported to induce the expression of *HSF3* and *HSP70* in the liver and spleen of broiler chickens after 5 days of exposure to CS, but TM mitigated this response [84]. During thermal stress, the decreased expression of heat shock proteins in TM chicks compared to controls suggests that TM chicks were less susceptible to thermal stress.

It could be concluded that the heat-induced expression of *HSP70* in the control chicks might be associated with *OCLN* expression. It was previously found that inhibiting *HSP* expression prevented an upregulation in *OCLN* expression *in vitro*, which led to a serious disturbance in the junctional localization of the *OCLN* protein during HS and resulted in high permeability of the tight junctions [85]. The current results show that, under TN conditions, TML chicks had significantly higher *HSP70* expression compared to controls. Previously, it was found that induction of *HSP70* was associated with acquired thermotolerance in different cell types [86].

### Impact of TML and TMH temperatures on the expression of inflammatory genes in broilers subjected to thermal stress

Deteriorated integrity of the tight junctions and intestinal barrier increases the paracellular permeability to luminal pathogens and toxic material, allowing them to move from the lumen and into the bloodstream [87]. This may result in an inflammatory response when these toxins or pathogens bind to TLRs (namely, *TLR2* and *TLR4*) and activate the signaling pathways, thereby inducing pro-inflammatory cytokines such as *IL6* and *IL8* [88-90]. *IL6* and *IL8* are pro-inflammatory cytokines with critical roles in innate immunity and the induction of the acute phase response [91,92]. *IL6* is a key regulator of both systematic and local acute inflammation primarily by modulating pro-inflammatory cytokine levels [93]. Several studies have found that acute HS causes an increase in *IL6* levels, which then triggers tissue protection mechanisms [82,94-97]. *IL6* promotes tissue repair by activating the *IL8* cytokine, which plays an important role in wound healing [92,98,99]. In fact, it has been hypothesized that, during acute HS, *IL6*, and its related pathways play a critical role in regulating the mechanisms that protect against tissue disruption in broiler chicken small yellow follicles [100].

*IL-6* acts as a heat-shock gene in chickens, being activated by *HSF3* during HS. Such regulation is required because increased *IL-6* expression in chicken yellow follicles in response to AHS has been linked to the regulation of protective pathway [101]. *IL-6* is required for tissue protection, healing, and regeneration in response to stress or tissue injury [102-104]. Indeed, pre-treatment with *IL-6* has been shown to have a protective effect during AHS exposure in mice by regulating physiological responses, resulting in tissue damage shielding [94]. This suggests that TM boosted the immune-protective response by ­increasing *IL-6* expression during HS. The previous studies reported that HS induces *TLR4*, *IL6*, and *IL8* expression in the small intestines of both chickens and mammals [14,95,105]. Furthermore, corticosterone-induced stress led to upregulated *IL6* and *IL8* expression in chickens [106,107].

In this study, thermal stress led to upregulation expression of *IL-6*, *IL-8*, *TLR2*, and *TLR4* in control incubation-group, and upregulated *IL-6*, *IL-8*, and *TLR4* in TMH subgroup in comparison with their TN counterparts. However, *TLR4* expression was significantly lower in TMH compared to control group during heat exposure. In TML incubation-group, the expression of *IL-6*, *TLR2*, and *TLR4* under thermal stress was comparable to that under TN condition, and their expression in TML was significantly lower than that in control group exposed to thermal stress. However, *IL-8* expression in TML was increased only under cold exposure. The upregulation of the aforementioned immune response genes might be triggered by the invasion of Gram-negative bacteria as a result of disrupted intestinal barrier [108], or by to the direct effect of the HS, since *TLR4* has been considered as a stress-related biosensor [109]. In addition, *TLR-4* activation can lead to intestinal barrier damage, since it was reported that *TLR-4* knockout mice were protected from intestinal hyperpermeability induced by burn [90]. The downregulated expression of immune response genes in TM chicks may indicate that the barrier damage in the ileum under thermal stress was attenuated in comparison with the control. Similarly, it was reported previously that HS led to the divergent expression of *TLR2*, *TLR4*, and other pro-inflammatory cytokines in the jejunum of TM chicks and chicks incubated under standard conditions [40].

TLRs are pathogen-recognition receptors, meaning they can recognize pathogen-associated signals as well as danger-associated molecular patterns (DAMPs) [110]. *HSP70*, a DAMP that is secreted by various cells during stress and binds to *TLR2* and *TLR4*, is one example [111,112]. The interaction of *HSP70* with *TLR2* and *TLR4* activates signal transduction cascades, which results in the activation of NF-B, which induces the expression of *IL-6* [111]. This points to a third mechanism for *IL-6* induction by DAMPs during HS: the activation of healing responses. Overall, the current findings suggest that TM improves broiler resistance to AHS by promoting tissue healing, ­regeneration, and protective immune responses characterized by the induction of *IL-6* expression and the components of its production pathways.

### Impact of TML and TMH temperatures on the histomorphology of the small intestine in broilers subjected to thermal stress

The major site of nutrient digestion and absorption in broilers is the small intestine, where the VH and VSA are important indicators of this intestinal function. Moreover, CD and the VH: CD ratio are especially important indicators of the cellular turnover at the villous surface. It was previously reported that longer villi and greater villous surface area suggest an enhanced nutrient absorption capability [113-115]. If the CD is deeper, this might indicate a rapid metabolism to maintain the intestinal villi renewal [113,115]. Any shortening in the VH or CD could result in a decreased nutrients absorption capability of the intestine [114,116].

The present study shows that thermal stress did not lead to significant histomorphometric changes in the ileum of TML and TMH chicks. However, in the control group, 7 days of thermal stress significantly increased the CD and decreased the VH: CD ratio in the ileum. Furthermore, heat exposure did not lead to significant histomorphometric changes in the jejunum of the different subgroups, while 4 days of CS led to significantly increased VH, VSA, and VH: CD ratio in the jejunum of both the TML and TMH groups, but it significantly increased the VH and CD in the control group. However, the TML and TMH subgroups had significantly increased VH: CD ratio compared to the control group after 4 days of CS.

In contrast, the previous studies reported a significant decrease in the VH and an increase in CD as a response to thermal stress, suggesting that thermal stress led to significant damage to the mucosal layer and the increased CD is a normal response to the renewal of the villi epithelia [21,23,24,117]. However, these studies were conducted with more extreme cold or heat exposure experiments in terms of temperature (the cold exposures were below 8°C) and duration (acute and prolonged/chronic exposures). On the other hand, early life cold acclimation and embryonic cold stimulation were found to enhance the metabolism and to improve the growth of the intestinal epithelia, which eventually led to increased VH [19,24,118]. In addition, continuous TM from embryonic day 11 until hatching day decreased the severity of post-hatch *S*. Enteritidis infection in the small intestine, since TM chicks possessed increased VH in the ileum and improved overall intestinal morphology [42]. Correspondingly, the results of the present study show that TM chicks had a higher VH: CD ratio compared to the control group after CS exposure.

## Conclusion

The present findings suggest that embryonic TM alleviated the impact of post-hatch thermal stress on the epithelial junctional barrier integrity and inflammatory response in the small intestine of broiler chickens. Furthermore, the current results may indicate that embryonic TM may alter the small intestine’s absorptive capability in broilers during post-hatch thermal stress exposure by increasing the VH and the CD. One limitation of the present study is that it had taken into consideration the mRNA transcription of genes but not the actual protein levels of junctional proteins. Moreover, further research is needed to understand the effect of more prolonged exposure to thermal stress and more extreme temperatures on the intestinal integrity of TM chicks.

## Authors’ Contributions

MBA: Funding acquisition and project administration. KEK and KMMS: Collection of the samples. KEK and KMMS: Data collection and analysis. MBA: Analyzed the data. All authors shared in writing of this manuscript and revised it. All authors read and approved the final manuscript.
